# RNA‐seq‐driven expression analysis to investigate cardiovascular disease genes with associated phenotypes among atrial fibrillation patients

**DOI:** 10.1002/ctm2.974

**Published:** 2022-07-25

**Authors:** Asude Berber, Habiba Abdelhalim, Saman Zeeshan, Sreya Vadapalli, Barr von Oehsen, Naveena Yanamala, Partho Sengupta, Zeeshan Ahmed

**Affiliations:** ^1^ Rutgers Institute for Health Health Care Policy and Aging Research Rutgers University New Brunswick New Jersey USA; ^2^ Rutgers Cancer Institute of New Jersey Rutgers University New Brunswick New Jersey USA; ^3^ Office of Advanced Research Computing, Rutgers The State University of New Jersey Computing Research and Education (CoRE) Building Piscataway New Jersey USA; ^4^ Division of Cardiovascular Disease Robert Wood Johnson Medical School Rutgers Biomedical and Health Sciences New Brunswick New Jersey USA; ^5^ Department of Medicine Robert Wood Johnson Medical School Rutgers Biomedical and Health Sciences New Brunswick New Jersey USA

To the Editor

Atrial fibrillation (AF) is defined as the high‐frequency excitation of the atrium, resulting in both dyssynchronous atrial contraction and the irregularity of ventricular excitation.^1^ According to its condition, AF disease is divided into two sub‐types: paroxysmal and persistent. In contrast to persistent AF, paroxysmal AF is diagnosed in the first phase of the disease, which later progresses to persistent AF.^1^ Furthermore, AF includes risk factors such as obesity, diabetes, smoking and a sedentary lifestyle and is prevalent in the older males of European ancestry. Previous studies have shown that both heart failure (HF) and cardiovascular diseases (CVD) contribute to an increased risk of AF.^1^ In this study, we investigated genes responsible for AF with sub‐disease groups through transcriptomic analysis (Additional file 1: High‐resolution figures). It was conducted as a continuation of our thorough CVD research focusing on HF performed on 61 CVD patients (Sample IDs: 1058–1118) and 10 patients without CVD (Control IDs: 648–658) (Additional file 2: population details). When grouped by gender and race, there were 40 males and 21 females, 42 Whites, 7 Blacks (Blacks or African Americans), 1 Asian, 1 Decline to Answer, 2 others, and 8 NA (Table 1 and Figure 1A). Peripheral blood samples were used for RNA extraction, and sequencing was performed using Illumina NovaSeq 6000‐S4 to assess the RNA quality.^2^ An efficient data management system (PROMIS‐LCR) with data extraction, transfer and loader system (ETL), created by the authors,^3^ was used for patient recruitment and consent tracking as well as dealing with the multi‐omics data, respectively.^4^ We also created a publicly available gene‐disease database, PAS‐Gen, which includes over 59 000 protein‐coding and non‐coding genes, and over 90 000 classified gene‐disease associations, to ease the gene‐disease visualization for researchers, medical practitioners and pharmacists.

**TABLE 1:** A total number of atrial fibrillation (AF) patient samples were used for the investigative study

**ID**	**Gender**	**Race**	**Age**	**Type**
648	Male	White	30	Control
649	Male	White	38	Control
650	Male	White	69	Control
651	Female	White	67	Control
652	Male	White	63	Control
653	Female	White	34	Control
655	Male	White	62	Control
656	Female	White	62	Control
657	Female	White	72	Control
658	Female	White	60	Control
1058	Female	White	72	Case
1059	Male	White	79	Case
1060	Male	NA	58	Case
1061	Male	White	70	Case
1062	Male	White	67	Case
1063	Male	White	66	Case
1064	Female	NA	54	Case
1065	Female	White	51	Case
1066	Male	White	82	Case
1067	Male	White	62	Case
1068	Male	NA	70	Case
1069	Female	White	65	Case
1070	Male	White	57	Case
1071	Female	Asian	52	Case
1072	Female	White	91	Case
1073	Female	White	89	Case
1074	Female	White	81	Case
1075	Female	White	59	Case
1076	Male	White	45	Case
1077	Male	White	73	Case
1078	Female	White	72	Case
1079	Male	NA	92	Case
1080	Male	White	86	Case
1081	Male	Black	57	Case
1082	Female	Black	59	Case
1083	Male	White	85	Case
1084	Female	Other	69	Case
1085	Male	Other	64	Case
1086	Male	Black	65	Case
1087	Female	NA	69	Case
1088	Female	White	65	Case
1089	Male	White	55	Case
1090	Male	White	70	Case
1091	Male	White	77	Case
1092	Male	White	62	Case
1093	Female	White	70	Case
1094	Male	White	64	Case
1095	Male	White	66	Case
1096	Male	Black	59	Case
1097	Female	White	57	Case
1098	Male	NA	83	Case
1099	Male	White	67	Case
1100	Male	NA	81	Case
1101	Male	White	64	Case
1102	Male	Black	71	Case
1103	Male	White	80	Case
1104	Male	White	73	Case
1105	Female	White	71	Case
1106	Male	NA	79	Case
1107	Male	White	84	Case
1108	Female	Black	57	Case
1109	Male	White	75	Case
1110	Male	Decline to Answer	80	Case
1111	Female	White	86	Case
1112	Male	White	72	Case
1113	Male	White	60	Case
1114	Female	Black	54	Case
1115	Male	White	67	Case
1116	Female	White	63	Case
1117	Male	White	66	Case
1118	Male	White	88	Case

*Note*: This table includes patient ID, gender (40 males and 21 females), age and race (42 White, 7 Black: Black or African American, 1 Asian, 1 declined to answer, and 8 NA). Samples 1058–1118 were obtained from CVD patients, whereas samples 648–658 were obtained from healthy individuals. The age of healthy individuals is not available.

Abbreviations: CVD, cardiovascular diseases; NA, not available.

**FIGURE 1:**
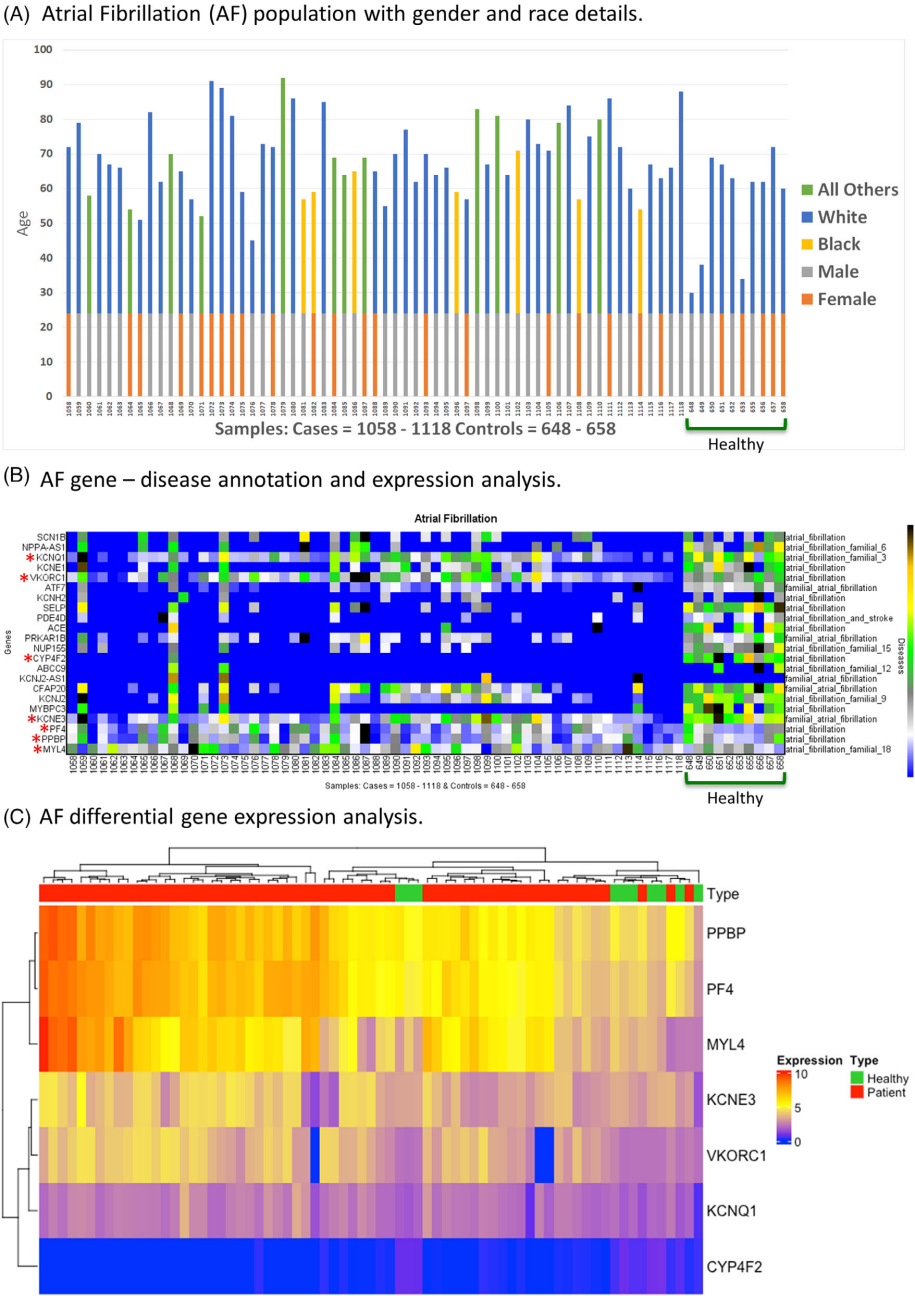
(A) Gender, age and race details of the atrial fibrillation (AF) population. (B) Gene‐disease annotation and expression analysis of all AF genes. (C) Differential expression analysis of AF gene. Gender, age and race information table for both patient and healthy control groups. The *X*‐axis signifies samples (AF ids: 1058–1118 and healthy ids: 648–658), and the *Y*‐axis indicates ages. The blue, yellow, grey and orange bars indicate both race and gender groups; White, Black, male and female, respectively. Genes‐disease heat map for the expression analysis of AF among all diseased and healthy control patients. The *X*‐axis signifies samples (AF ids: 1058–1118 and healthy ids: 648–658), the left *Y*‐axis shows genes, and the right *Y*‐axis presents genes associated with the AF. Differential gene expression heat map of AF for all patients and healthy control groups

First, the transcriptomic data analysis involved the development of an RNA‐seq processing pipeline that contained four operating parts: (I) data pre‐processing, (II) data quality checking, (III) data storage and management and (IV) data visualization (Additional file 1: High‐resolution figures).^2^ The analysis of transcripts per million (TPM) was performed to normalize the RNA‐seq data by using the visualizing genes with disease‐causing variants environment with the findable, accessible, intelligent and reproducible approach (Additional file 4: AF analysis ‐ gene expression data). It reveals all genes annotated with their associated clinical AF phenotype using gene–disease association.^2^, ^5^ This expression analysis was expanded to visualize the classification of protein‐ and non‐coding genes in detail as gender‐ and race‐based. First, we looked across the AF‐annotated genes to identify protein‐ and non‐coding genes together and found 71 genes related to AF and relative diseases (Additional file 3: Complete Gene List). Next, we observed expression in protein‐coding genes and found 22 genes associated with direct and relative AF diseases, which are denominated as AF phenotypes (*SCN1B, NPPA‐AS1, KCNQ1, KCNE1, VKORC1, ATF7, KCNH2, SELP, PDE4D, ACE, PRKAR1B, NUP155, CYP4F2, ABCC9, KCNJ2‐AS1, CFAP20, KCNJ2, MYBPC3, KCNE3, PF4, PPBP, MYL4*) (Figure 1B and Table 2). After the initial analysis, differential gene expression analysis was implemented to further investigate AF genes. Of the protein‐coding genes, seven AF‐associated genes (*MYL4, PPBP, PF4, KCNE3, VKORC1, KCNQ1* and *CYP4F2*) showed differentially regulated expression (Figure 1C). A previous study has reported some of these genes (*GJA5*, *KCNA5, KCNE2, KCNJ2, KCNQ1, KCNH2, NPPA* and *SCN5A*) as novel genes for familial AF in the absence of mutations, whereas mutations in *MYL4* have been strongly associated with AF disease in humans.^6^

**TABLE 2:** List of genes associated with atrial fibrillation (AF) diseases

**ENSEMBL ID**	**Gene name**	**Category**	**Disease**
ENSG00000105711	SCN1B	Protein coding	Atrial fibrillation
ENSG00000242349	NPPA‐AS1	Antisense/non‐coding	Atrial fibrillation familial 6
ENSG00000053918	KCNQ1	Protein coding	Atrial fibrillation familial 3
ENSG00000180509	KCNE1	Protein coding	Atrial fibrillation
ENSG00000167397	VKORC1	Protein coding	Atrial fibrillation
ENSG00000170653	ATF7	Protein coding	Familial atrial fibrillation
ENSG00000055118	KCNH2	Protein coding	Atrial fibrillation
ENSG00000174175	SELP	Protein coding	Atrial fibrillation
ENSG00000113448	PDE4D	Protein coding	Atrial fibrillation and stroke
ENSG00000159640	ACE	Protein coding	Atrial fibrillation
ENSG00000188191	PRKAR1B	Protein coding	Familial atrial fibrillation
ENSG00000113569	NUP155	Protein coding	Atrial fibrillation familial 15
ENSG00000186115	CYP4F2	Protein coding	Atrial fibrillation
ENSG00000069431	ABCC9	Protein coding	Atrial fibrillation familial 12
ENSG00000267365	KCNJ2‐AS1	Antisense/non‐coding	Familial atrial fibrillation
ENSG00000070761	CFAP20	Protein coding	Familial atrial fibrillation
ENSG00000123700	KCNJ2	Protein coding	Atrial fibrillation familial 9
ENSG00000134571	MYBPC3	Protein coding	Atrial fibrillation
ENSG00000175538	KCNE3	Protein coding	Familial atrial fibrillation
ENSG00000163737	PF4	Protein coding	Atrial fibrillation
ENSG00000163736	PPBP	Protein coding	Atrial fibrillation
ENSG00000198336	MYL4	Protein coding	Atrial fibrillation familial 18

*Note*: This table includes the ENSG ID, gene name, category and disease associated with that gene.

With a deeper investigation of the normalized expression analysis, we found that *PF4, PPBP, MYL4, KCNE3, VKORC1, KCNQ1* and *CYP4F2* genes are highly expressed in AF (Figure 1B) with relative diseases as AF phenotypes; AF (both for *PF4* and *PPBP*); AF familial 18; familial AF, AF, AF familial 3, AF, respectively (Additional file 5: Information about AF phenotypes). The phenotypes represent different subsets of how the disease presents when it is inherited based on the gene of interest. Additionally, these findings were supported by another study in which two long non‐coding RNAs genes were found to interact with protein‐coding genes associated with AF.^7^ A subsequent analysis was performed based on two groupings: race‐ and gender‐based. The race‐based analysis involved Black, White and all other races in which *PF4, PPBP* and *MYL4* were found to be highly expressed protein‐coding genes in AF in all different race groups (Figure 2A–C). Although *KCN3* appeared in the analysis, it did not show consistent expression across the patients. In addition, the *PPBP* gene, which is one of the three immune‐related genes (*CXCL12, CCL4*), has been found to have a positive relationship with the infiltration of immune cells (e.g. neutrophils, plasma cells and resting dendritic cells) and plays a role in the development of AF disease.^8^ Furthermore, the gene expression analysis based on gender segregation showed similar results, with *PF4*, *PPBP* and *MYL4* genes as highly expressed with AF disease in both female and male groups (Figure 2D,E).

**FIGURE 2:**
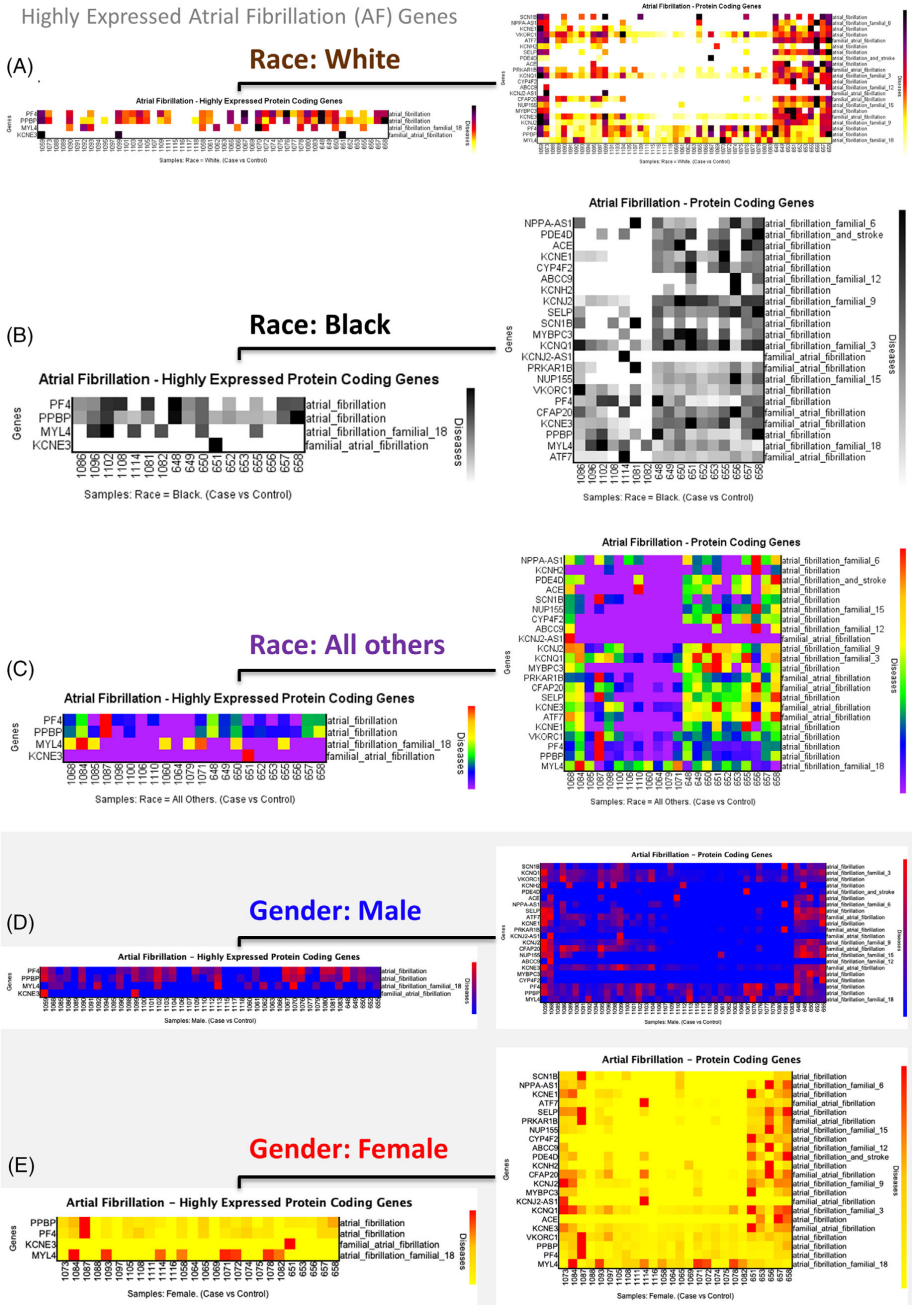
Race‐ and gender‐based gene expression analysis of atrial fibrillation (AF) genes. All and highly expressed protein‐coding genes related to AF in self‐described Whites (A), Blacks/African Americans (B), and all other races (C), and male (D) and female (E)

In summary, we performed the systematic transcriptomic characterization of AF‐associated genes. Our findings report three highly expressed genes and their associated diseases as AF phenotypes; *PF4*: AF; *PPBP*: AF and *MYL4*: AF familial 18, with a similar expression pattern across races and genders. Moreover, when we compared the genes associated with HF from our previous CVD/HF study^2^ with those associated with AF, we discovered that two genes (*ACE* and *MYBPC3*) were associated with both diseases (HF and AF). These findings are valuable for future research studies as they signify the potential to further investigate these genes for mutations and disease‐specific variants. This will provide a new path focusing on a more personalized approach to therapy and treatment. In the future, we seek to evaluate the causal basis for AF by moving beyond the one gene‐one disease model through the integration of the expressed genome, characterization of mutations derived from genomic signatures and mapping them on phenotypic traits in the electronic medical records. We aim to contribute to the paradigm shift in the application and interpretation of genetic and genomically informed medicine for AF, moving from a deterministic conceptualization to a probabilistic interpretation of genetic risk. This will support diagnostic and preventive care delivery strategies beyond traditional symptom‐driven, disease‐causal medical practice. We aim to construct machine learning models to identify a baseline transcriptional signature highly predictive of response across these indications.^9^ This might accelerate our ability to leverage and extend the information contained within the original data and to model patient‐specific genomics and clinical data for significant transcriptional correlations, highlighting the association of genetic variants to clinical outcomes of treatment in AF and other CVD.^5^, ^9^, ^10^

## CONFLICT OF INTEREST

The authors declare no conflict of interests regarding financial or non‐financial aspects.

## Supporting information

Supplementary Material 1: High‐resolution figures.

Supplementary Material 2: Population details.

Supplementary Material 3: Detailed genes list.

Supplementary Material 4: AF analysis – gene expression data.

Supplementary Material 5: Information about AF phenotypes.
