# Supplementary material for: RNA‐seq‐driven expression analysis to investigate cardiovascular disease genes with associated phenotypes among atrial fibrillation patients
Source: Clin Transl Med. 2022 Jul 25;12(7):e974. doi: 10.1002/ctm2.974 (PMC9309637; doi:10.1002/ctm2.974)
Supplement: Supplementary file 1 — Supplementary Material 1: High‐resolution figures. [file CTM2-12-e974-s001.pdf]

Supplementary Material 1: High Resolution Figures

Title

RNA-seq driven expression analysis to investigate Cardiovascular disease genes with associated phenotypes among Atrial Fibrillation patients

Running Head

Gene expression analyses of CVD/AF patients

Authors

Asude Berber[1, †], Habiba Abdelhalim[1, †], Saman Zeeshan[2], Sreya Vadapalli[1], Barr von Oehsen[3], Naveena Yanamala[4], Partho Sengupta[4], and Zeeshan Ahmed[1, 5, *]

Affiliations

1. Rutgers Institute for Health, Health Care Policy and Aging Research, Rutgers University, 112 Paterson St, New Brunswick, NJ, USA.
2. Rutgers Cancer Institute of New Jersey, Rutgers University, 195 Little Albany St, New Brunswick, NJ, USA.
3. Office of Advanced Research Computing, Rutgers, The State University of New Jersey, Computing Research and Education (CoRE) Building, 96 Frelinghuysen Road, Room 704, Piscataway, NJ, USA.
4. Division of Cardiovascular Disease, Robert Wood Johnson Medical School, Rutgers Biomedical and Health Sciences, 125 Paterson St, New Brunswick, NJ, USA.
5. Department of Medicine, Robert Wood Johnson Medical School, Rutgers Biomedical and Health Sciences, 125 Paterson St, New Brunswick, NJ, USA.

[†]Equally contributing first authors.

[*]Corresponding author(s): Zeeshan Ahmed (zahmed@ifh.rutgers.edu)

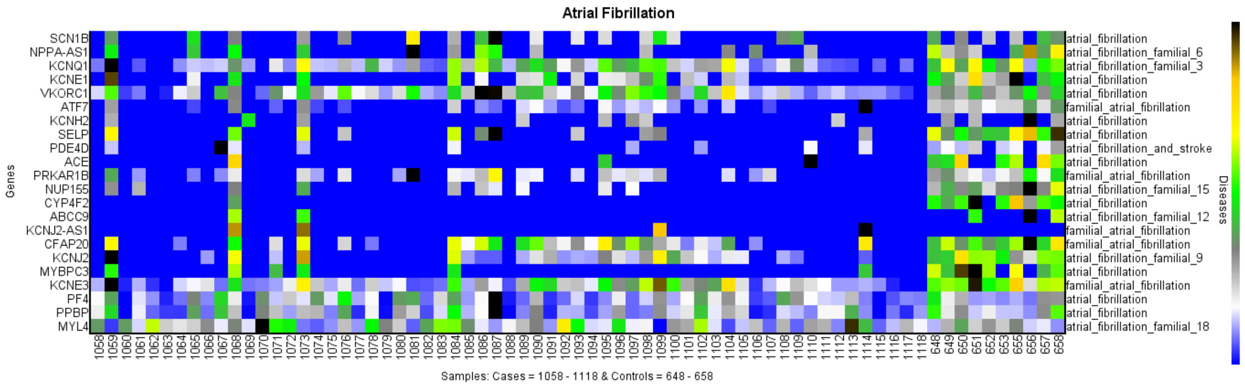

Supplementary Figure 1: All expressed genes related to Atrial Fibrillation (AF).

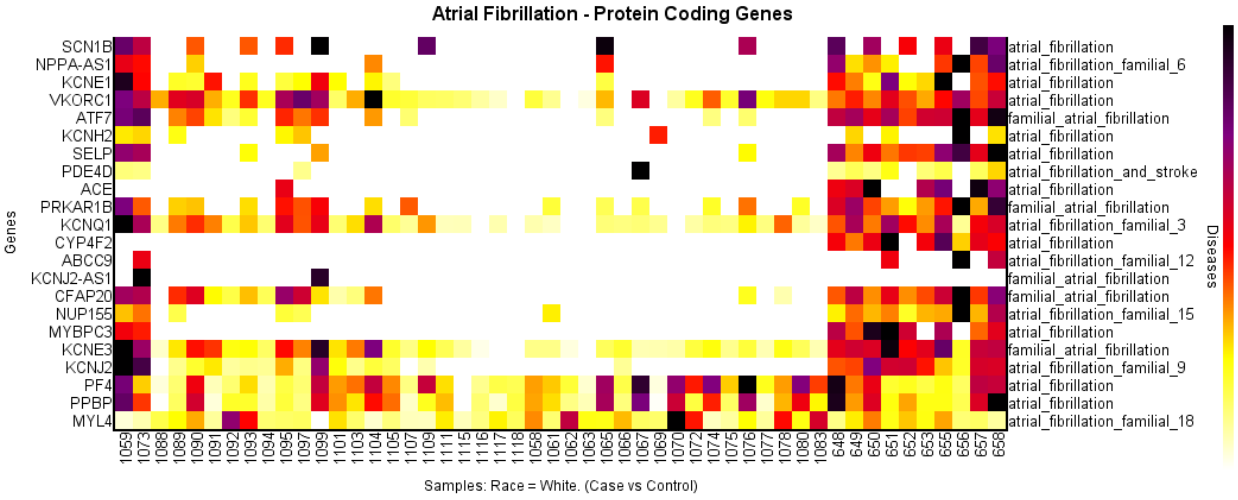

Supplementary Figure 2: All protein-coding genes related to AF in self-described Whites.

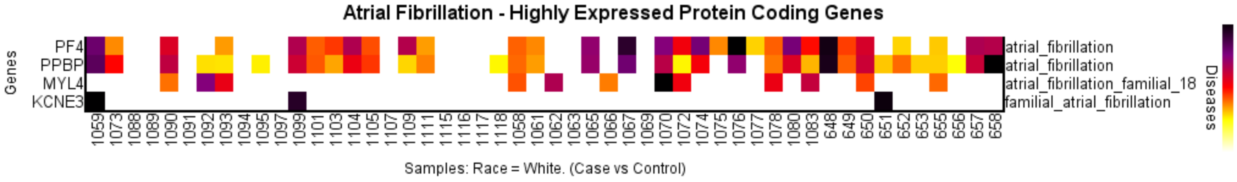

Supplementary Figure 3: Highly expressed protein-coding genes related to AF in self-described Whites.

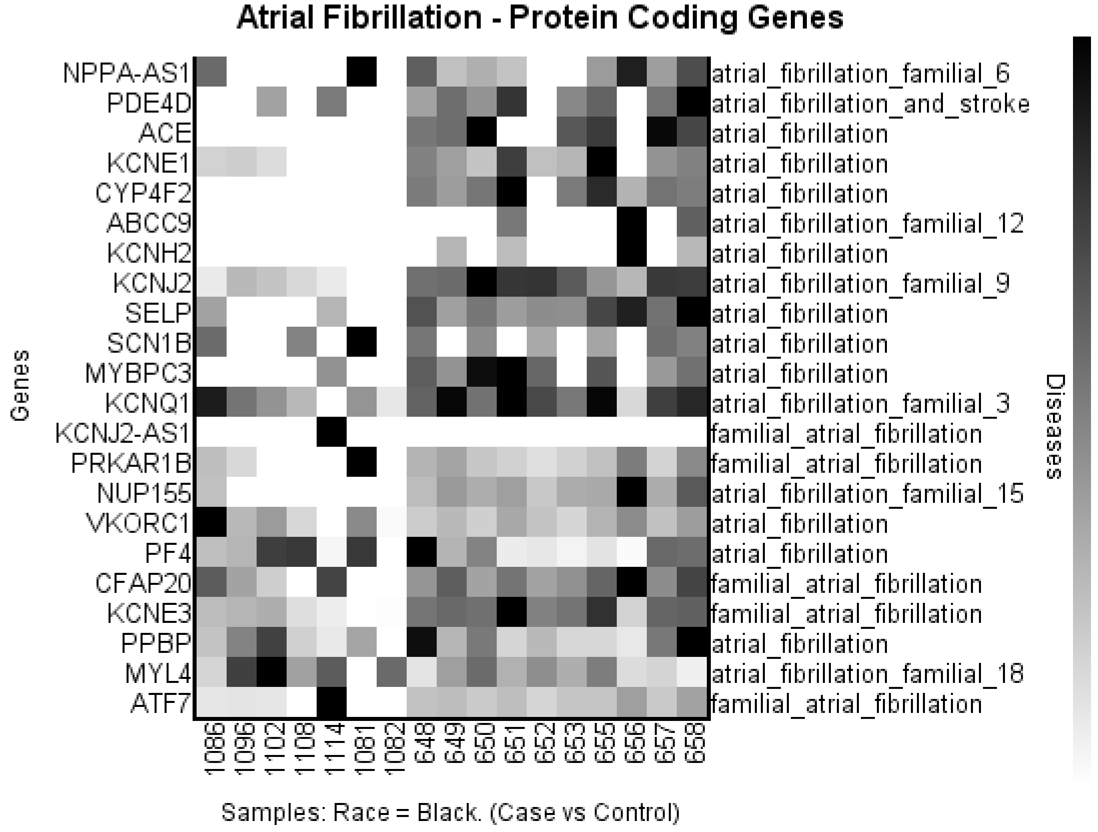

Supplementary Figure 4: All protein-coding genes related to AF in self-described Blacks/African Americans.

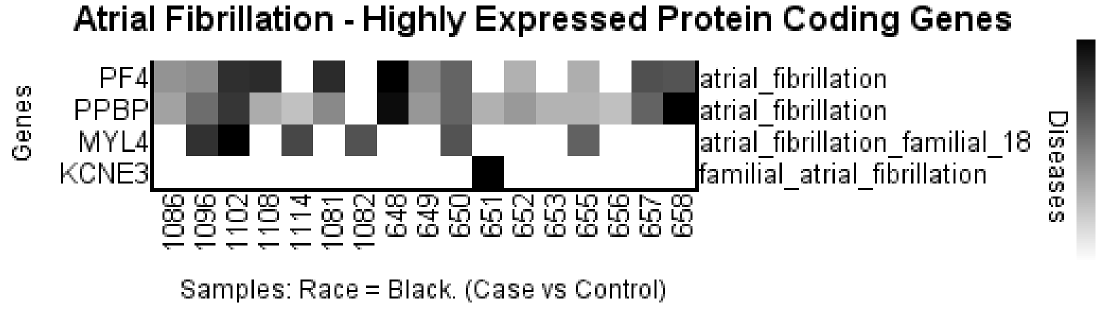

Supplementary Figure 5: Highly expressed protein-coding genes related to AF in self-described Blacks/African Americans.

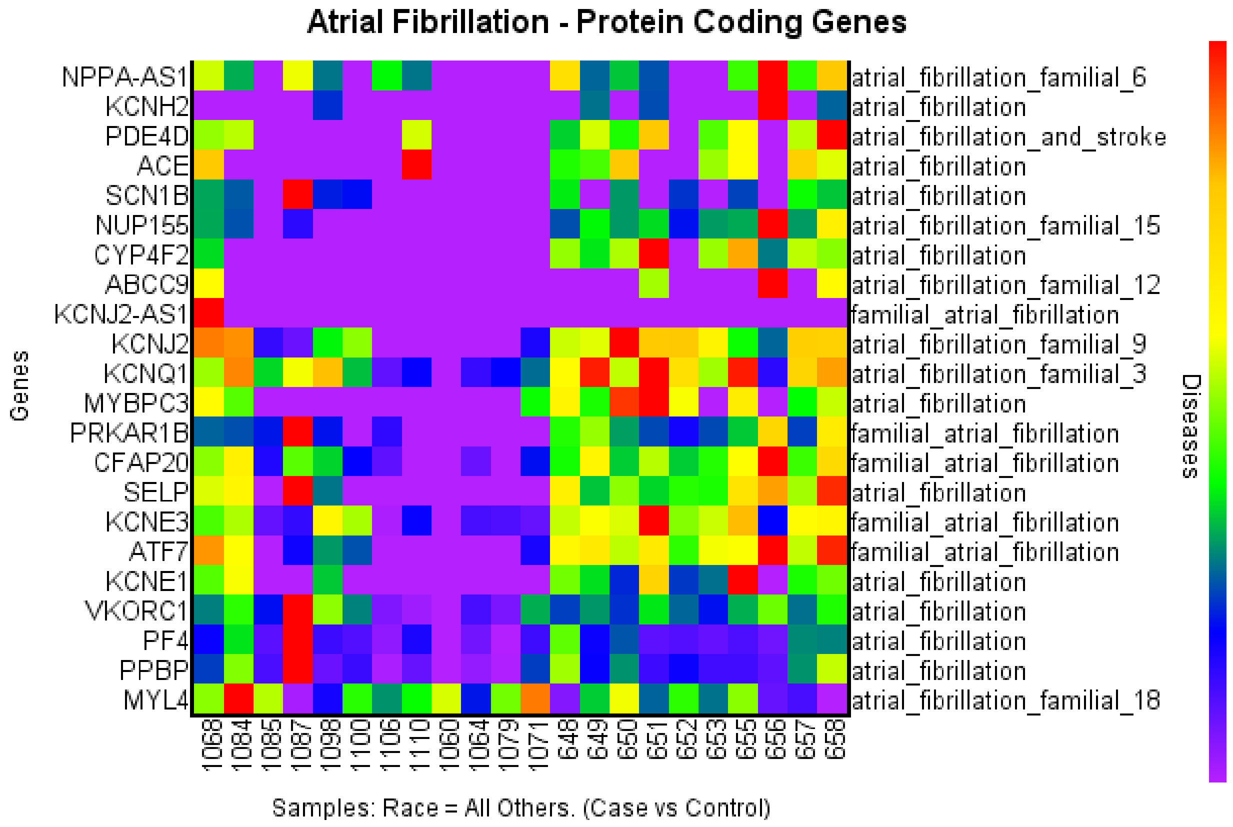

Supplementary Figure 6: All protein-coding genes related to AF in self-described other races.

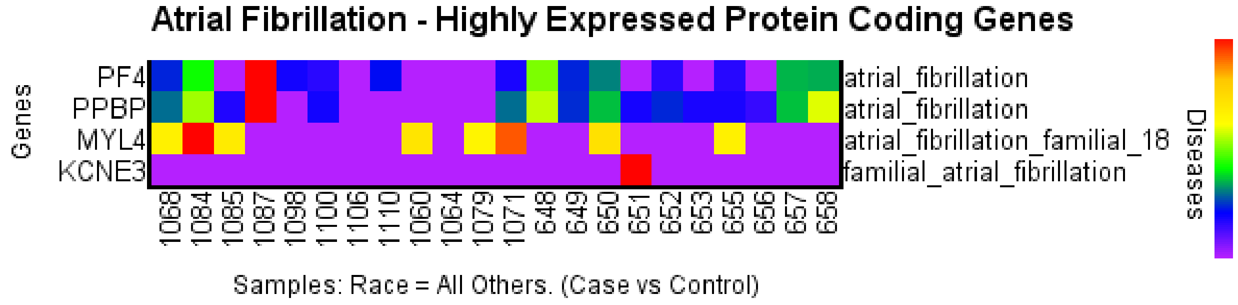

Supplementary Figure 7: Highly expressed protein-coding genes related to AF in self-described other races.

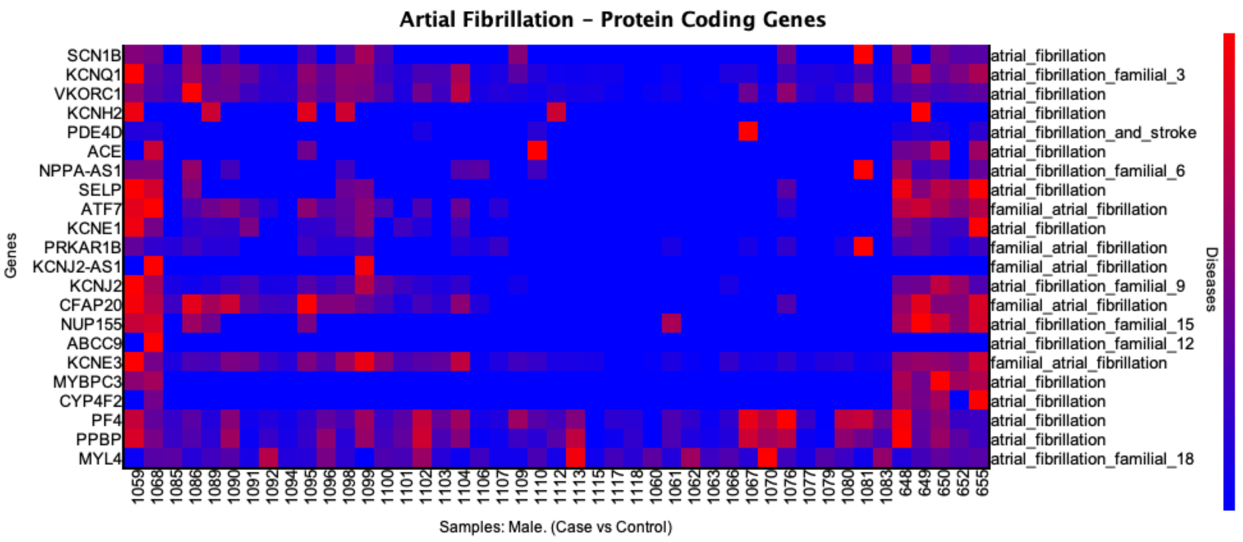

Supplementary Figure 8: All protein-coding genes related to AF in Males.

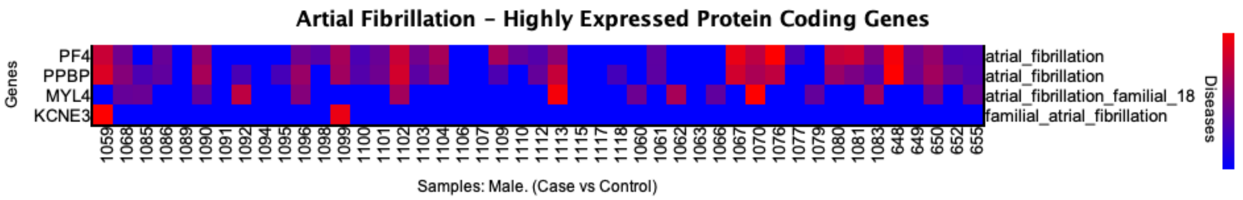

Supplementary Figure 9: Highly expressed protein-coding genes related to AF in Males.

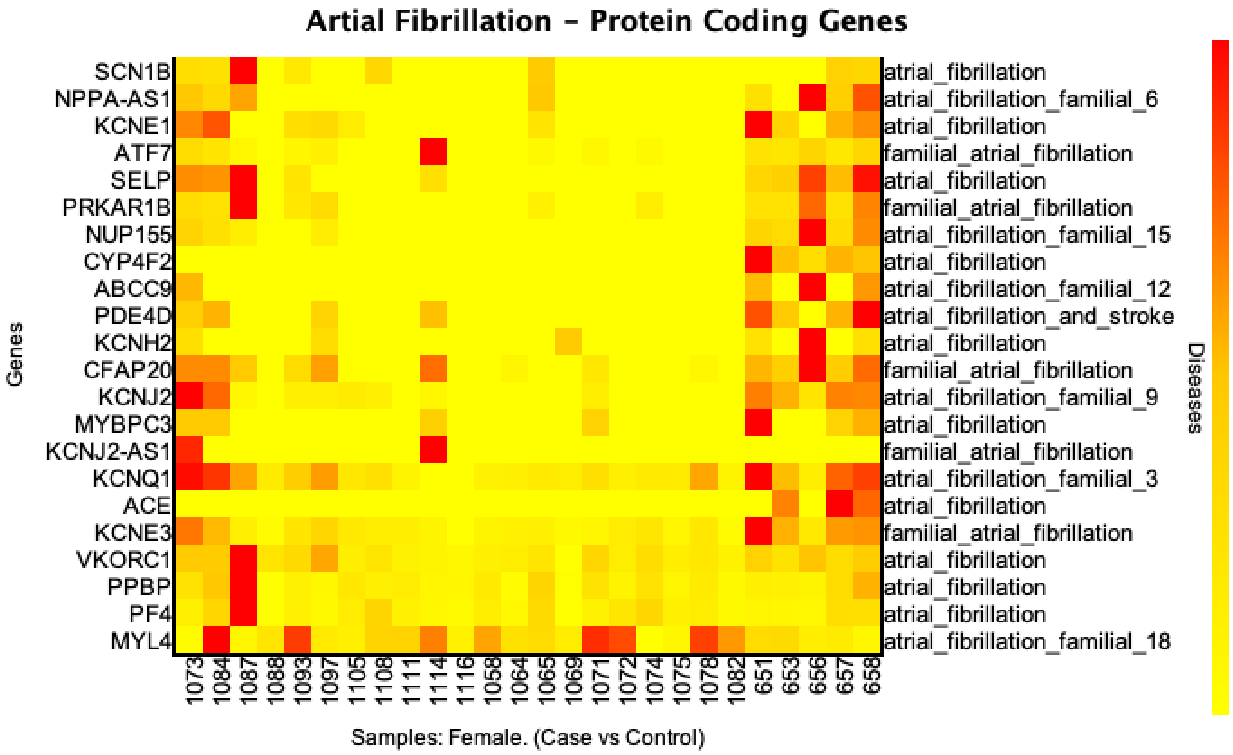

Supplementary Figure 10: All protein-coding genes related to AF in Females.

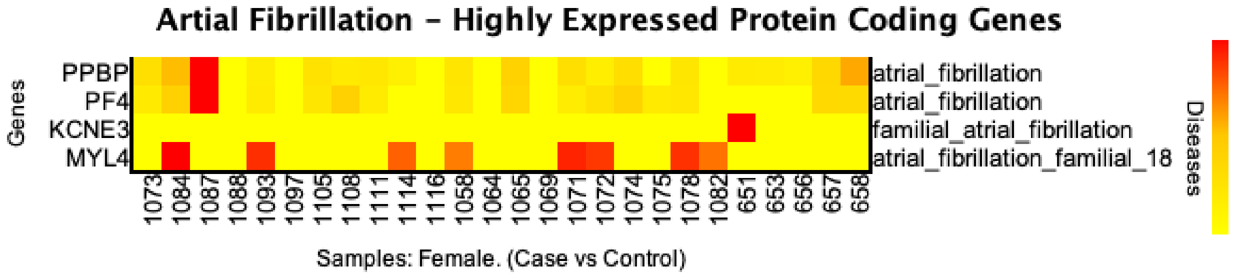

Supplementary Figure 11: Highly expressed protein-coding genes related to AF in Females.

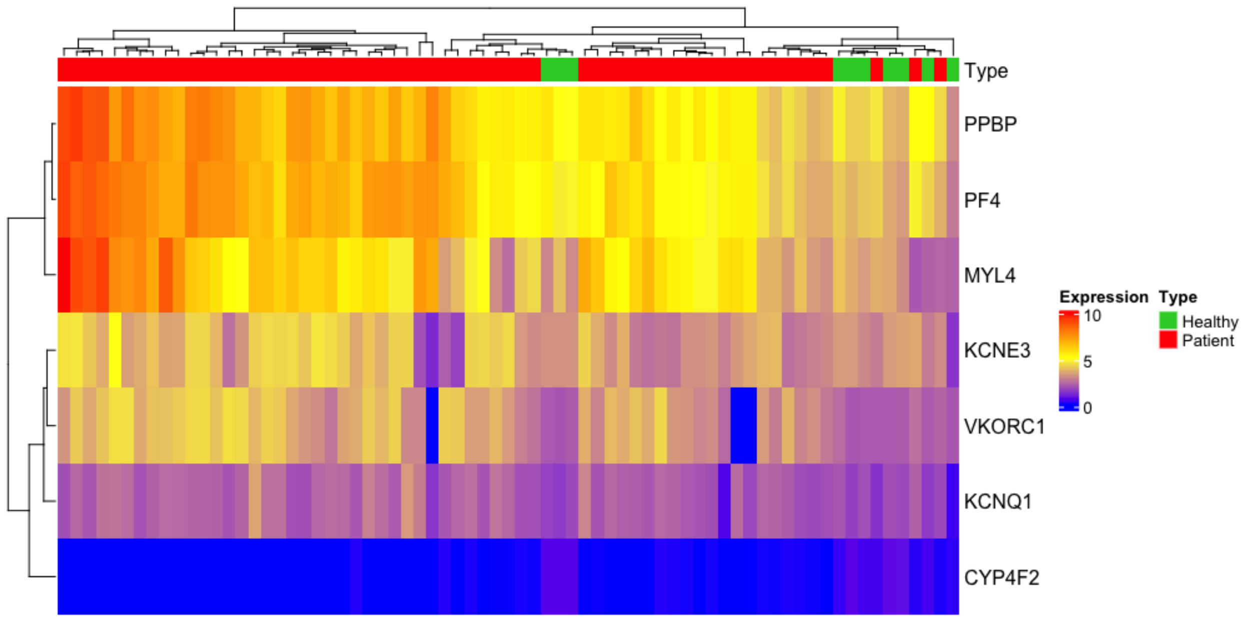

Supplementary Figure 12: Differential expression analysis of AF genes.

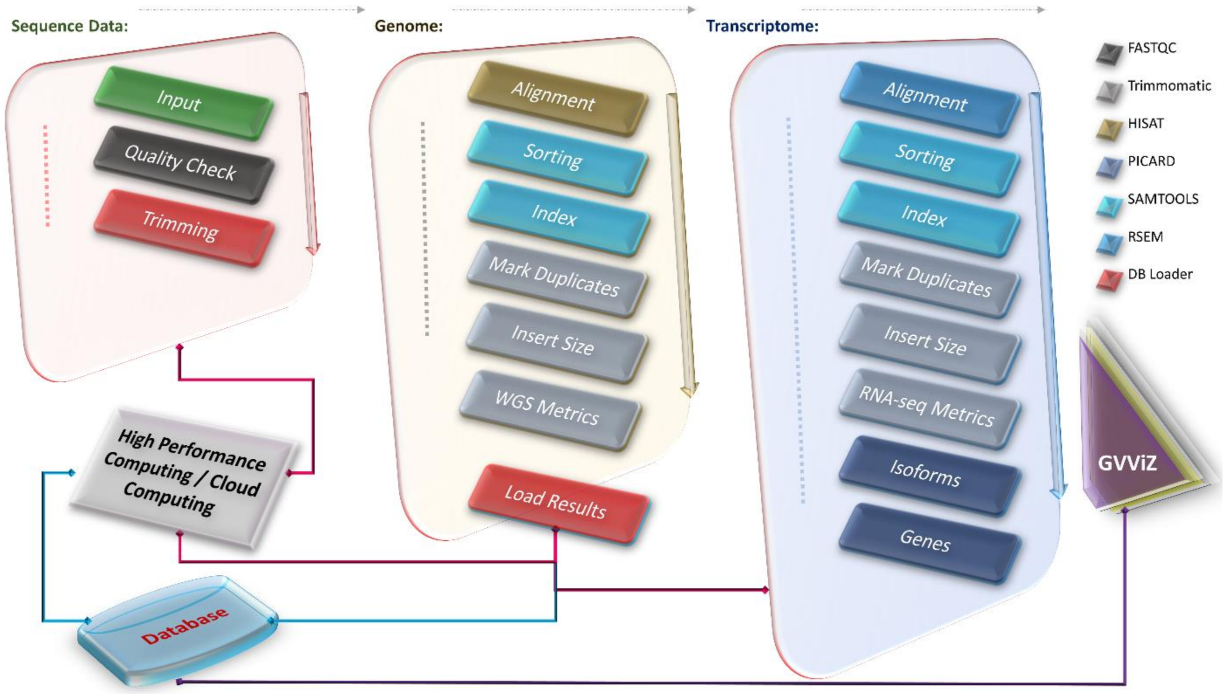

Supplementary Figure 13: Bioinformatics pipelines for RNA-seq data processing, and expression and enrichment analysis. It includes quality checking (QC), remove adapters and low-quality sequences, sort, and index sequences, remove duplicates, compute size distribution, and read orientation of paired-end libraries, align sequences to the human reference genome, quantify and identify differentially expressed genes, and annotation, analysis, and visualization with GVViZ.
