# Supplementary material for: RNA‐seq‐driven expression analysis to investigate cardiovascular disease genes with associated phenotypes among atrial fibrillation patients
Source: Clin Transl Med. 2022 Jul 25;12(7):e974. doi: 10.1002/ctm2.974 (PMC9309637; doi:10.1002/ctm2.974)
Supplement: Supplementary file 5 — Supplementary Material 5: Information about AF phenotypes. [file CTM2-12-e974-s002.pdf]

Supplementary Material 5: Information about AF phenotypes

Title

RNA-seq driven expression analysis to investigate cardiovascular disease genes with associated phenotypes among Atrial Fibrillation patients

Running Head

Gene expression analyses of CVD/AF patients

Authors

Asude Berber[1, †], Habiba Abdelhalim[1, †], Saman Zeeshan[2], Sreya Vadapalli[1], Barr von Oehsen[3], Naveena Yanamala[4], Partho Sengupta[4], and Zeeshan Ahmed[1, 5, *]

Affiliations

1. Rutgers Institute for Health, Health Care Policy and Aging Research, Rutgers University, 112 Paterson St, New Brunswick, NJ, USA.
2. Rutgers Cancer Institute of New Jersey, Rutgers University, 195 Little Albany St, New Brunswick, NJ, USA.
3. Office of Advanced Research Computing, Rutgers, The State University of New Jersey, Computing Research and Education (CoRE) Building, 96 Frelinghuysen Road, Room 704, Piscataway, NJ, USA.
4. Division of Cardiovascular Disease, Robert Wood Johnson Medical School, Rutgers Biomedical and Health Sciences, 125 Paterson St, New Brunswick, NJ, USA.
5. Department of Medicine, Robert Wood Johnson Medical School, Rutgers Biomedical and Health Sciences, 125 Paterson St, New Brunswick, NJ, USA.

[†]Equally contributing first authors.

[*]Corresponding author(s): Zeeshan Ahmed (zahmed@ifh.rutgers.edu)

Information about AF phenotypes:

Atrial Fibrillation (AF) is defined as frequent atrium excitation that results in both irregular atrial convulsion and ventricular impulse. It is a precursor to increased risk factors of ischemic stroke and systemic embolism in patients with preexisting heart failure (HF) disease [**S1, S2, S3**]. In addition, previous studies reported that both heart failure (HF) and other cardiovascular diseases (CVDs) contribute to an increased risk of AF [**S4, S5**].

Genetic variant evaluation is playing a vital role in the predisposition of AF. According to the published literature, many common and rare variants (protein-coding sequence regions) have been associated with AF and its family's phenotypes [**S6**, **S7, S8**]. It has been reported that AF may occur due to the genetic tendency, especially, when considering some young age patients with AF [**S6**]. Likewise, in our study we were focused on analyzing gene expression, and disease causing variants that may give rise to a variety of AF familial phenotypes. To support clinical and translational research, we were able to match most of the AF diseases (discussed in this study) with the International Classification of Diseases (ICD) code: **"**427.31" (version 9) and "I48.91" (version 10). ICD codes used are maintained by the World Health Organization (WHO) [**S9**]. However, we have utilized variable public human gene-disease annotation databases to link genes with relevant AF disease codes [**S10, S11, S12**].

Conditionally, AF disease has been reported to be distributed among three sub-types: **I)** paroxysmal, **II)** persistent, and **III)** long-standing persistent (permanent) [**S1, S13**]. Paroxysmal AF is generally diagnosed in the first phase of the disease. It progresses to persistent AF, throughout the disease condition [**S1, S3, S13**]. However, some studies have indicated that the relationship between paroxysmal and persistent AF is not decisive. In contrast, another study reported that paroxysmal AF remains unchanged, while persistent AF can spontaneously lead to paroxysmal AF [**S1**]. An alternative study conducted on the pathophysiological link between HF and AF has reported that the long-standing sub-type of AF is the most frequently diagnosed type of AF. Furthermore, differentiating patients with both HF and AF, and AF only; persistent AF has been found to be the most widely spread, since these two diseases have the similar underlying pathophysiology [**S4**].

Abbreviations

Atrial Fibrillation (AF)

Heart Failure (HF)

Cardiovascular diseases (CVD)

International Classification of Diseases (ICD)

World Health Organization (WHO)

References

S1. Staerk, L., Sherer, J. A., Ko, D., Benjamin, E. J., & Helm, R. H. (2017). Atrial Fibrillation: Epidemiology, Pathophysiology, and Clinical Outcomes. Circulation research, 120(9), 1501–1517. https://doi.org/10.1161/CIRCRESAHA.117.309732.

S2. Amin, A., Houmsse, A., Ishola, A., Tyler, J., & Houmsse, M. (2016). The current approach of atrial fibrillation management. Avicenna journal of medicine, 6(1), 8–16. https://doi.org/10.4103/2231-0770.173580

S3. Wijesurendra, R. S., & Casadei, B. (2019). Mechanisms of atrial fibrillation. Heart (British Cardiac Society), 105(24), 1860–1867. https://doi.org/10.1136/heartjnl-2018-314267

S4. Carlisle, M. A., Fudim, M., DeVore, A. D., & Piccini, J. P. (2019). Heart Failure and Atrial Fibrillation, Like Fire and Fury. JACC. Heart failure, 7(6), 447–456. https://doi.org/10.1016/j.jchf.2019.03.005

S5. Kwok, M. K., & Schooling, C. M. (2021). Mendelian randomization study on atrial fibrillation and cardiovascular disease subtypes. Scientific reports, 11(1), 18682. https://doi.org/10.1038/s41598-021-98058-w

S6. Brundel, B., Ai, X., Hills, M. T., Kuipers, M. F., Lip, G., & de Groot, N. (2022). Atrial fibrillation. Nature reviews. Disease primers, 8(1), 21. https://doi.org/10.1038/s41572-022-00347-9

S7. Fatkin, D., Santiago, C. F., Huttner, I. G., Lubitz, S. A., & Ellinor, P. T. (2017). Genetics of Atrial Fibrillation: State of the Art in 2017. Heart, lung & circulation, 26(9), 894–901. https://doi.org/10.1016/j.hlc.2017.04.008

S8. Assum, I., Krause, J., Scheinhardt, M. O., Müller, C., Hammer, E., Börschel, C. S., Völker, U., Conradi, L., Geelhoed, B., Zeller, T., Schnabel, R. B., & Heinig, M. (2022). Tissue-specific multi-omics analysis of atrial fibrillation. Nature communications, 13(1), 441. https://doi.org/10.1038/s41467-022-27953-1

S9. Fung, K. W., Xu, J., & Bodenreider, O. (2020). The new International Classification of Diseases 11th edition: a comparative analysis with ICD-10 and ICD-10-CM. Journal of the American Medical Informatics Association: JAMIA, 27(5), 738–746. https://doi.org/10.1093/jamia/ocaa030

S10. Zeeshan, S., Xiong, R., Liang, B. T., & Ahmed, Z. (2020). 100 Years of evolving gene-disease complexities and scientific debutants. Briefings in bioinformatics, 21(3), 885–905. https://doi.org/10.1093/bib/bbz038

S11. Ahmed, Z., Zeeshan, S., Mendhe, D., & Dong, X. (2020). Human gene and disease associations for clinical-genomics and precision medicine research. Clinical and translational medicine, 10(1), 297–318. https://doi.org/10.1002/ctm2.28

S12. Ahmed, Z., Zeeshan, S., Xiong, R., & Liang, B. T. (2019). Debutant iOS app and gene-disease complexities in clinical genomics and precision medicine. Clinical and translational medicine, 8(1), 26. https://doi.org/10.1186/s40169-019-0243-8

S13. Sagris, M., Vardas, E. P., Theofilis, P., Antonopoulos, A. S., Oikonomou, E., & Tousoulis, D. (2021). Atrial Fibrillation: Pathogenesis, Predisposing Factors, and Genetics. International journal of molecular sciences, 23(1), 6. https://doi.org/10.3390/ijms23010006

Acknowledgments

We appreciate great support by the Rutgers Institute for Health, Health Care Policy, and Aging Research (IFH); Department of Medicine, Rutgers Robert Wood Johnson Medical School (RWJMS); and Rutgers Biomedical and Health Sciences (RBHS), at the Rutgers, The State University of New Jersey. We thank members and collaborators of Ahmed Lab at the Rutgers (IFH, RWJMS, RBHS) for their support, participation, and contribution to this study.
